# The Novel Peptide AEDPPE Alleviates Trophoblast Cell Dysfunction Associated With Preeclampsia by Regulating the NF-κB Signaling Pathway

**DOI:** 10.3389/fcvm.2021.738378

**Published:** 2021-12-17

**Authors:** Yixiao Wang, Yan Cao, Xiaohong Ji, Ting Li, Lu Xue, Chanjuan Li, Ruizhe Jia, Hongjuan Ding

**Affiliations:** Women's Hospital of Nanjing Medical University, Nanjing Maternity and Child Health Care Hospital, Nanjing, China

**Keywords:** peptide, preeclampsia, trophoblast, dysfunction, NF-κB pathway

## Abstract

**Background:** Preeclampsia (PE) is a serious risk to the health of pregnant women and fetuses during pregnancy, and there is no effective treatment for this condition. Although many reports have confirmed the therapeutic effects of peptides in diseases, the role of peptides in PE remains poorly understood.

**Methods:** A differentially expressed peptide in PE (AEDPPE) is derived from heat-shock protein beta-1 (HSPB1), amino acids 100 to 109 (DVNHFAPDEL), which we identified in a previous study. We synthesized AEDPPE and investigated its effect on HTR-8/SVneo cell function using a Cell Counting Kit-8, flow cytometric assay, and Transwell and wound-healing assays. Quantitative reverse transcription-PCR and ELISA were used to determine cytokine expression. Pull-down assay, mass spectrometry, Western blot analysis, and immunofluorescence were used to explore the potential targets and signaling pathways regulated by AEDPPE. Finally, we assessed the effect of AEDPPE in the lipopolysaccharide (LPS)-induced PE-like rat model.

**Results:** AEDPPE significantly promoted the migration and invasion of HTR-8/SVneo cells, and it decreased the expression of interleukins 1 beta (IL-1β), interleukin 6 (IL-6), and interleukin 8 (IL-8). These functions performed by AEDPPE remained evident after injury to HTR-8/SVneo cells with tumor necrosis factor-alpha (TNF-α), and AEDPPE reversed the elevated sFlt-1/PlGF ratio induced by TNF-α. AEDPPE may exert these biological effects by binding to heat-shock protein 90β (HSP 90β) and, thus, affect the NF-κB signaling pathway. In an LPS-induced PE-like rat model, AEDPPE significantly improved PE symptoms and fetal rat outcomes.

**Conclusion:** Our study showed that AEDPPE enhanced trophoblast migration and invasion and reduced inflammatory cytokine expression, and we hypothesized that these actions involved the NF-κB signaling pathway. The use of AEDPPE may thus develop into a novel modality in the treatment of PE.

## Background

Preeclampsia (PE) is a systemic disease with or without proteinuria that occurs for the first time after 20 weeks of pregnancy, affecting 3–5% of pregnant women (1). The pathogenesis of PE involves inflammation (2), oxidative stress (3), and the renin-angiotensin system (4). PE can affect almost every organ and system, and thus leads to various adverse complications such as placental abruption and fetal growth restriction (5).

Although the pathogenesis of PE is presently unclear, it is widely recognized to primarily involve two stages: insufficient trophoblast invasion of the spiral arteries through the uterine meconium that causes inadequate placental perfusion and systemic inflammation followed by extensive systemic endothelial injury (1, 6, 7). Trophoblast cells invade the endometrium during normal pregnancy, causing remodeling of the spiral arteries so as to increase the blood and nutritional supplies of the placenta (8, 9). However, in patients with PE, the decline in trophoblast invasion results in insufficient remodeling of the spiral arteries, and the blood supply of the placenta diminishes, leading to systemic inflammatory reactions and endothelial dysfunction (2, 10, 11). Recent studies have shown that the expression of soluble FMS-like tyrosine kinase 1 (sFlt-1), placental growth factor (PlGF), and other factors also changes; and that these factors play an important role in different pathogenic processes (12, 13). In addition to these factors, tumor necrosis factor-α (TNF-α); interleukins 1 beta (IL-1β), 6 (IL-6), and 8 (IL-8); and other inflammation-related factors are increased in the serum of patients with PE (14, 15). These factors are extremely important in the pathogenesis and prediction of PE. However, PE remains an intractable condition. Prophylactic low-dose aspirin reduces the risk of preterm delivery in PE; but once PE is diagnosed, there is no curative treatment other than the delivery of the fetus. As no drugs have been shown to affect disease progression (16), there is an urgent need for such pharmaceutical agents in the treatment of PE.

A recent study has revealed that peptides can no longer be simply regarded as amino acid chains but rather as bioactive substances. Peptide research involves many fields, such as investigations of tumors (17), diabetes (18), and infections (19). A polypeptide from the junction region sequence of EWS-fli 1 inhibits cell cloning, cell-cycle kinetics, and the uptake of bromodeoxyuridine in Ewing's sarcoma cells (20). Antimicrobial peptides (AMPs) from tilapia also manifest an antitumor effect by destroying the microtubular network in cells (21). In our previous study, we found that peptides derived from the serum of patients with PE significantly protected against endothelial dysfunction in umbilical vein vessels (22, 23). Thus, the exploration of peptides with therapeutic properties for PE may provide novel candidates for the future treatment of PE.

In a previous study, we analyzed the differences in peptide content in the placenta of patients with PE and healthy pregnant women by peptidomic analysis (24), and we uncovered a differentially expressed peptide in PE (AEDPPE) that increased the migration and invasion of HTR-8/SVneo cells and downregulated the expression of inflammatory factors. We subsequently explored a role for AEDPPE and its underlying mechanism in trophoblast dysfunction and then investigated the *in vivo* effects of AEDPPE on lipopolysaccharide (LPS)-induced PE-like model in rats.

## Materials and Methods

### The Peptides

AEDPPE is located at amino acid positions 100 to 109 (DVNHFAPDEL) of heat-shock protein beta-1 (HSPB1). AEDPPE was synthesized by Shanghai Science Peptide Biological Technology Co., Ltd., with a purity of >95%. The carrier peptide of the HIV TAT protein (GRKKRRQRRPPQQ) was covalently attached to the N-terminal end of AEDPPE to increase the efficiency of peptide entry into the cell and to enhance its biological activity (25, 26). AEDPPE was dissolved in suitable double-distilled water at concentrations of 1, 5, 10, and 20 μM for the experiments. N-terminal isothiocyanate fluorophore-labeled AEDPPE (FITC-AEDPPE) and N-terminal biotin-labeled AEDPPE (biotin-AEDPPE) were also chemically synthesized with a purity of >95%. FITC-AEDPPE was used at a concentration of 10 μM. FITC-AEDPPE was co-incubated with HTR-8/SVneo cells for 1 h to observe the intracellular distribution of AEDPPE.

### Cell Culture and Treatment

The immortalized human first-trimester trophoblast cell-line HTR-8/SVneo cells (ATCC, Rockville, MD, USA) were grown in RPMI 1640 medium (Gibco, Rockville, MD, USA) containing 10% fetal bovine serum (FBS; Gibco, Rockville, MD, USA) in a humid environment containing 5% CO_2_ and 95% air at 37°C. Different concentrations of AEDPPE (1, 5, 10, and 20 μM) were used to stimulate HTR-8/SVneo cells for 24 h. For the treatment of HTR-8/SVneo cells, TNF-α obtained from Sigma-Aldrich (T6674-10UG; St. Louis, MO, USA) was dissolved in double-distilled water, and TNF-α at 20 ng/ml was used to induce inflammation and dysfunction of HTR-8/SVneo cells (27–30).

### Cell Counting Kit-8 Assay

The HTR-8/SVneo cells (3 × 10^3^ cells/well) were pre-cultured in 96-well plates in 5% CO_2_ at 37°C, and then, these cells were treated with different concentrations of AEDPPE or TNF-α. Furthermore, 10 μl of Cell Counting Kit-8 (CCK-8, Dojindo, Kumamoto, Japan) was added to each well at 0, 24, 48, and 72 h. After incubation of the cells in the incubator for 2 h, the absorbance of each well at a wavelength of 450 nm was measured by a multifunctional microplate reader (Hybrid Technology™, BioTek, Winooski, VT, USA).

### Wound-Healing Assay

When HTR-8/SVneo cells were cultured to full fusion in a six-well plate, wounds were created with the tip of a 200-μl pipette, and three spots were randomly marked. Six-well plates were rinsed with phosphate-buffered saline (PBS) until dead cells were completely removed, and then, 2 ml of RPMI 1640 medium (Gibco, Rockville, MD, USA) without FBS was added. Different concentrations of AEDPPE or TNF-α were added to the culture medium. Images were taken at the marker sites at 0, 24, and 48 h after culture and analyzed by the ImageJ software (National Institutes of Health, Bethesda, MD, USA) to determine the wound area and calculate the wound healing rate. The wound healing rate was the difference between the scratch area at the 24-h or 48-h time point and the scratch area at the 0-h time point divided by the scratch area at the 0-h time point (31).

### Cell Migration Assay and Invasion Assay

#### Cell Migration Assay

A total of 30,000 HTR-8/SVneo cells and 200 μl of RPMI 1640 medium without FBS were added to the upper chamber of the Transwell (Costar, cat. no. 3422, Corning Inc., Corning, NY, USA), and 700 μl of RPMI 1640 medium containing 10% FBS was added to the lower chamber. Different concentrations of AEDPPE were added to the upper cavity and cultured in an incubator for 48 h with or without TNF-α. After the culture medium in the upper chamber was removed, 700 μl paraformaldehyde at a concentration of 4% was added, and the samples were fixed for 30 min. The cells were dyed with 0.5% crystal violet dye solution for 30 min in the dark, the cells and excess dye solution that have not passed through the polycarbonate membrane in the upper chamber were wiped away with cotton swabs, and then, the chamber was dried. Cells penetrating the polycarbonate membrane were photographed using a Zeiss inverted microscope (Zeiss, Oberkochen, Germany), and the number of cells was counted by the ImageJ software (National Institutes of Health, Bethesda, MD, USA).

#### Transwell Invasion Assay

Before the experiment, RPMI 1640 culture medium and Matrigel matrix glue (Corning Inc., Corning, NY, USA) were fully mixed in a volume of 1:7, and then, 60 μl of the mixture was added to the upper chamber of the Transwell and incubated overnight. Other experimental steps were the same as those in the cell migration assay.

### Quantitative Reverse Transcription PCR

Total RNA of HTR-8/SVneo cells was extracted by TRIzol reagent. According to the reagent instructions of the manufacturer, cDNA was synthesized by using HiScript II QRT Super Mix for qPCR (Vazyme, Jiangsu, China). Primers used in quantitative reverse transcription-PCR (qRT-PCR) were designed by Primer 5.0 (Premier, Canada) or other relevant literature and were blasted in NCBI. The expression level of all target genes was standardized to that of glyceraldehyde 3-phosphate dehydrogenase (GAPDH). qRT-PCR was performed by a Viia7 real-time PCR System (Applied Biosystems, Life Technologies, Carlsbad, CA, USA). The relative RNA expression was calculated by 2^−ΔΔCT^. The primer sequences were as follows: human IL-1β, 5′-CCAGGGACAGGTATGGAGCA-3′ (forward) and 5′-TTCAACACGCAGGACAGGTACAG-3′ (reverse); human IL-6, 5′-CTCAATATTAGAGTCTCAACCCCCA-3′ (forward), and 5′-GTGGGGCGGCTACATCTTT-3′ (reverse); human IL-8, 5′-CTTGGCAGCCTTCCTGATTT-3′ (forward) and 5′-AACCCTCTGCACCCAGTTTT-3′ (reverse); human sFlt-1, 5′-TTTGCCTGAAATGGTGAGTAAGG-3′ (forward) and 5′-CTTCCCAGCAAATCCTTCGGG-3′ (reverse); human PlGF, 5′-GAGACCCACAGACTGCCAC-3′ (forward) and 5′-ACCTTGGCCGGAAAGAACAA-3′ (reverse); human MMP-2, 5′-CCCCAGACAGGTGATCTTGAC-3′ (forward) and 5′-GCTTGCGAGGGAAGAAGTTG-3′ (reverse); human GAPDH, 5′-GGAGCGAGATCCCTCCAAAAT-3′ (forward), and 5′-GGCTGTTGTCATACTTCTCATGG-3′ (reverse).

### Cell Apoptosis Assay

Cell apoptosis was assessed by using the flow cytometry assay (BD, Franklin Lakes, NJ, USA). HTR-8/SVneo cells were stimulated by AEDPPE or TNF-α for 24 h and re-suspended in 100 μl binding buffer. Cells were then stained with Annexin V-FITC and propidium iodide (PI) for 15 min in the dark. Apoptosis was then measured using flow cytometry.

### Enzyme-Linked Immunosorbent Assay

Venous blood was collected from the inferior vena cava of rats on day 20 of gestation, and the serum was collected after centrifugation of venous blood at 2,000 × *g* for 20 min. HTR-8/SVneo cells were treated for 24 h, and the supernatant was collected after centrifugation at 2,000 × *g* for 20 min. The levels of rat TNF-α, IL-6, sFlt-1, PlGF, and human IL-6, sFlt-1, PlGF were measured according to the instructions of ELISA Kit (MLBIO, Shanghai, China).

### Pull-Down Assay and Mass Spectrometry

The samples from the AEDPPE group and control group were placed in 30 μl of Dynabeads™ M-280 Streptavidin (Thermo Fisher Scientific, Waltham, MA, USA) in a 1.5-ml Eppendorf (EP) tube and placed on a magnetic rack. After the liquid was clear, the supernatant was removed, 1 ml of premixed RIPA and PMSF (100:1) was added to an EP tube, and the sample was mixed well and placed on a magnetic rack. The AEDPPE group was treated with 200 μg biotin-AEDPPE, whereas the control group was treated with scrambled peptide and shaken overnight at 4°C. After the samples were washed with a mixed solution six times, a proper amount of protein solution of HTR-8/SVneo cells collected in advance was added, and the sample was shaken at 4°C overnight and then washed six times. Then, 20 μl of the mixed solution of RIPA buffer and PMSF and 5 μl of 1 × SDS were added. Appropriate amounts of samples were taken from each group for electrophoresis. Silver staining was carried out according to the instruction of Pierce Silver Stain Kit for Mass Spectroscopy (Thermo Fisher Scientific, Waltham, MA, USA), and the final bands were photographed. Different bands were cut out and analyzed by mass spectrometry analysis provided by BGI (Shenzhen, China). The target protein was screened preliminarily by Gene Ontology (GO) annotation analysis.

### Western Blotting

The HTR-8/SVneo cells were treated with TNF-α and AEDPPE for 24 h. The cells were washed twice with PBS, and 60 μl containing a mixture of RIPA lysate (Beyotime, Shanghai, China), benzene methyl sulfonyl fluoride (Beyotime, Shanghai, China), and phosphorylated protease inhibitor (SericeBio, Wuhan, China) was added to each well to lyse the cells thoroughly on ice. The BCA Protein Assay Kit (Beyotime, Shanghai, China) was used to measure the concentration of protein solution. Proteins (25 μg) were subjected to electrophoresis with a 10% polyacrylamide gel and transferred to a polyvinylidene fluoride membrane, which was blocked at room temperature for 2 h. The diluted primary antibody was incubated with the membrane at 4°C overnight. The membrane was washed with Tris-buffered saline Tween (TBST) on a shaker for 10 min each time, three times in total. The membrane was incubated with the corresponding secondary antibody at room temperature for 1 h and washed three times. Visualization of the immunoblots was performed by chemiluminescent reagents (WBKLS0500, Merck, Kenilworth, NJ, USA), and the grayscale values of the immunoblots were semi-quantified using the ImageJ software (National Institutes of Health, Bethesda, MD, USA).

The antibodies used in the study were as follows: IKK α (1:1,000, ab178870, Abcam, Cambridge, MA, USA), phosphorylated (p)-IKK α/β (Ser176/180, 1:1,000, 2697T, Cell Signaling Technology, Danvers, MA, USA), IκB α (1:1,000, ab32518, Abcam), p-IκBα (phospho S36, 1:10,000, ab133462, Abcam), NF-κB p65 (1:1,000, ab32536, Abcam), p-NF-κB p65 (phospho S536, 1:2,000, ab86299, Abcam), MMP-2 (1:1,000, ab97779, Abcam), HSP 90β (1:5,000, ab203085, Abcam), β-actin (1:2,500, ab8227, Abcam), goat anti-rabbit IgG H&L (HRP) (1:2,000, ab205718, Abcam), and goat anti-mouse IgG H&L (HRP) (1:2,000, ab205719, all the above antibodies are from Abcam, Cambridge, MA, USA).

### Immunofluorescence

The HTR-8/SVneo cells were collected and plated in a 24-well plate and pretreated with AEDPPE and TNF-α for 24 h. The cells were washed with preheated PBS, fixed with 4% paraformaldehyde at room temperature for 15 min, and treated with 0.5% Triton X-100 for 15 min. After the samples were blocked with goat serum for 30 min, a diluted antibody (1:200) was added overnight. The fluorescent secondary antibody (1:500, ab150081, Abcam, Cambridge, MA, USA) was incubated for 2 h at room temperature in the dark. After the addition of the secondary antibody, PBS washes were performed, 4′,6-diamidino-2-phenylindole (DAPI, 1:1,000, D1306, Thermo Fisher Scientific, Waltham, MA, USA) was added, and the cells were incubated for 5 min in the dark. DAPI was rinsed away, PBS was added, and the images were taken with a fluorescence microscope (Zeiss, Oberkochen, Germany).

### Animal Experiment

The animal experiment scheme was approved by the animal ethics committee of Nanjing Medical University (Approval No. IACUC-2005040), and all animal experiments met the ethical requirements of animal experiments. Sprague-Dawley (SD) rats were purchased from Viton Lihua Company (Beijing, China), weighing ~300 g and aged ~12 weeks. All rats were raised in a suitable environment. When the rats were acclimated to the environment, male and female adult rats were mated at a ratio of 1:2. The presence of sperm in the vaginal smear was used to confirm the success of maternal pregnancy and was recorded as gestational Day 0 (G0).

A PE-like rat model was constructed according to previously published studies with a little modification (32–34). All pregnant rats were randomly divided into three groups (*n* = 6 per group): normal saline group rats were injected with normal saline *via* the tail vein on the 5th day of pregnancy, the rats in the LPS group and LPS+AEDPPE group were injected with LPS (0.5 μg/kg) *via* the tail vein on the 5th day of pregnancy, and the rats in the LPS+AEDPPE group were injected with AEDPPE diluted with normal saline (5 mg/kg) *via* the tail vein on the 11, 14, and 17th days of pregnancy. On the 20th day, the pregnant rats were euthanized, and the placenta and fetal rats were weighed. Rat kidneys and placentas were collected for histological analysis, kidney slices were stained for hematoxylin–eosin (H&E), and sections from the placental labyrinth were stained for H&E and periodic acid-Schiff (PAS).

The systolic blood pressure of the rat tail artery was measured on the 4, 7, 15, 17, and 19th days of pregnancy by a BL series biological function experimental system (Chengdu Taimeng Software Co., Ltd., Chengdu, China), and the average value was calculated after the systolic pressure of each rat was measured three times. Urine samples of rats were collected for 24 h on the 4, 7, and 19th days of pregnancy and centrifuged at 2,000 rpm at room temperature. The supernatant was collected, and the total amount of urine protein was measured by a urine protein test kit (Nanjing Jiancheng Bioengineering Institute, Nanjing, China).

### Statistical Analysis

The results of the present study are presented as the mean ± SD. Statistical analyses were performed by using the GraphPad Prism 7 software (San Diego, CA, USA). Significant differences between the two groups were analyzed using Student's *t*-test, and a *p*-value < 0.05 was considered a statistically significant result.

## Results

### Characterization of Peptide AEDPPE

**Figure 1:**
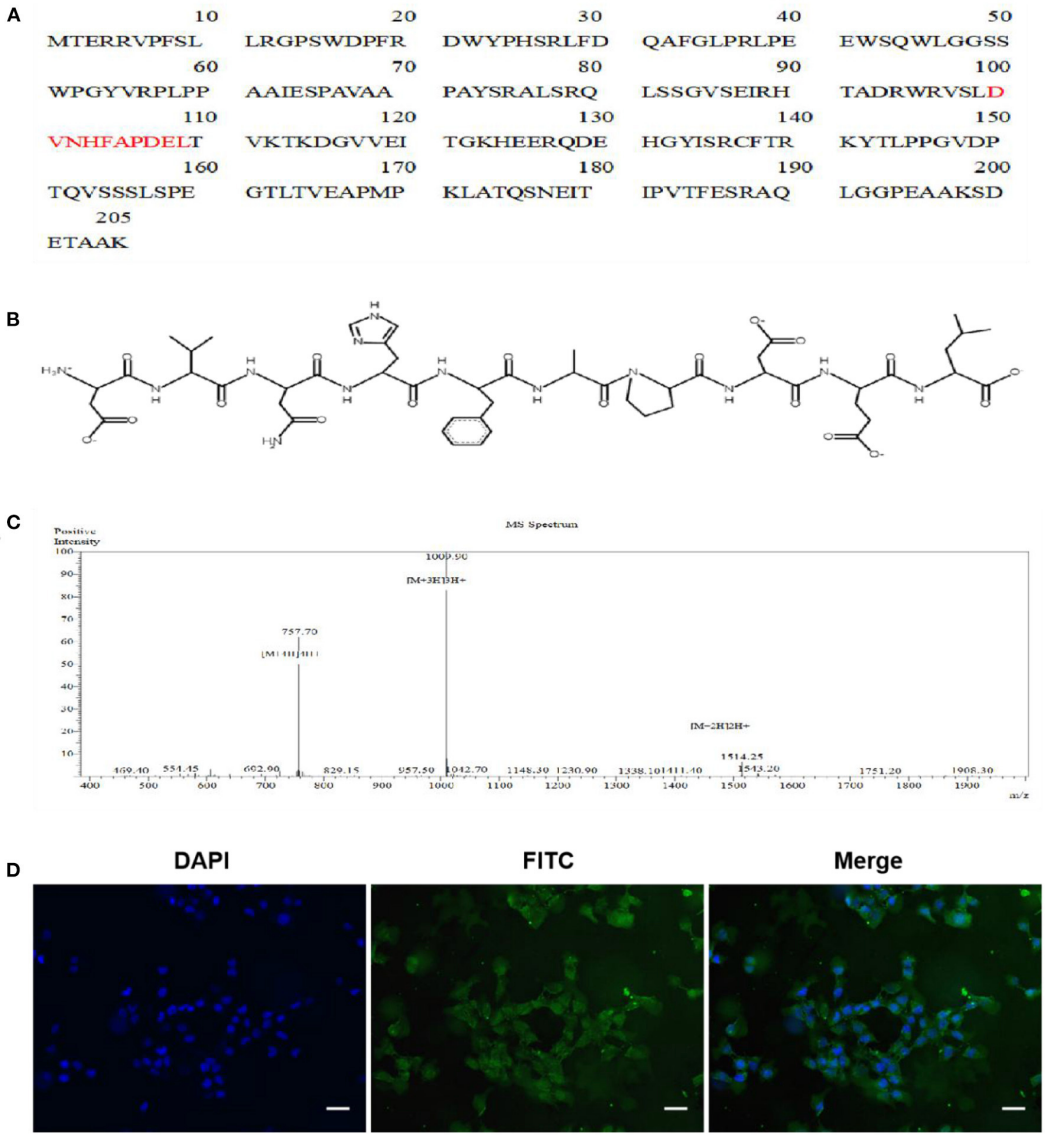
Characterization of peptide AEDPPE. **(A)** AEDPPE is located at amino acids 100 to 109 of heat-shock protein beta-1 (HSPB1). **(B)** Chemical structure of AEDPPE. **(C)** Mass spectral analysis of chemically synthesized AEDPPE. **(D)** HTR-8/SVneo cells were incubated with FITC-AEDPPE (green) for 1 h and then stained with DAPI (blue). AEDPPE was widely distributed throughout the cytoplasm and nucleus after penetrating the cell membrane (scale bar = 100 μm).

The AEDPPE is a peptide containing 10 amino acids from HSPB1 with an average molecular weight of 1,156.19 g/mol (Figure 1A). The chemical structure and synthetic mass spectra of AEDPPE are shown in Figures 1B,C, respectively. To investigate whether AEDPPE can cross the cell membrane, FITC-labeled AEDPPE was synthetized and added to the culture medium of HTR-8/SVneo cells. We found that AEDPPE crossed the cell membrane and was distributed in both the nucleus and cytoplasm (Figure 1D).

### AEDPPE Downregulates the Production of Inflammatory Factors and Promotes Migration and Invasion of HTR-8/SVneo Cells

**Figure 2:**
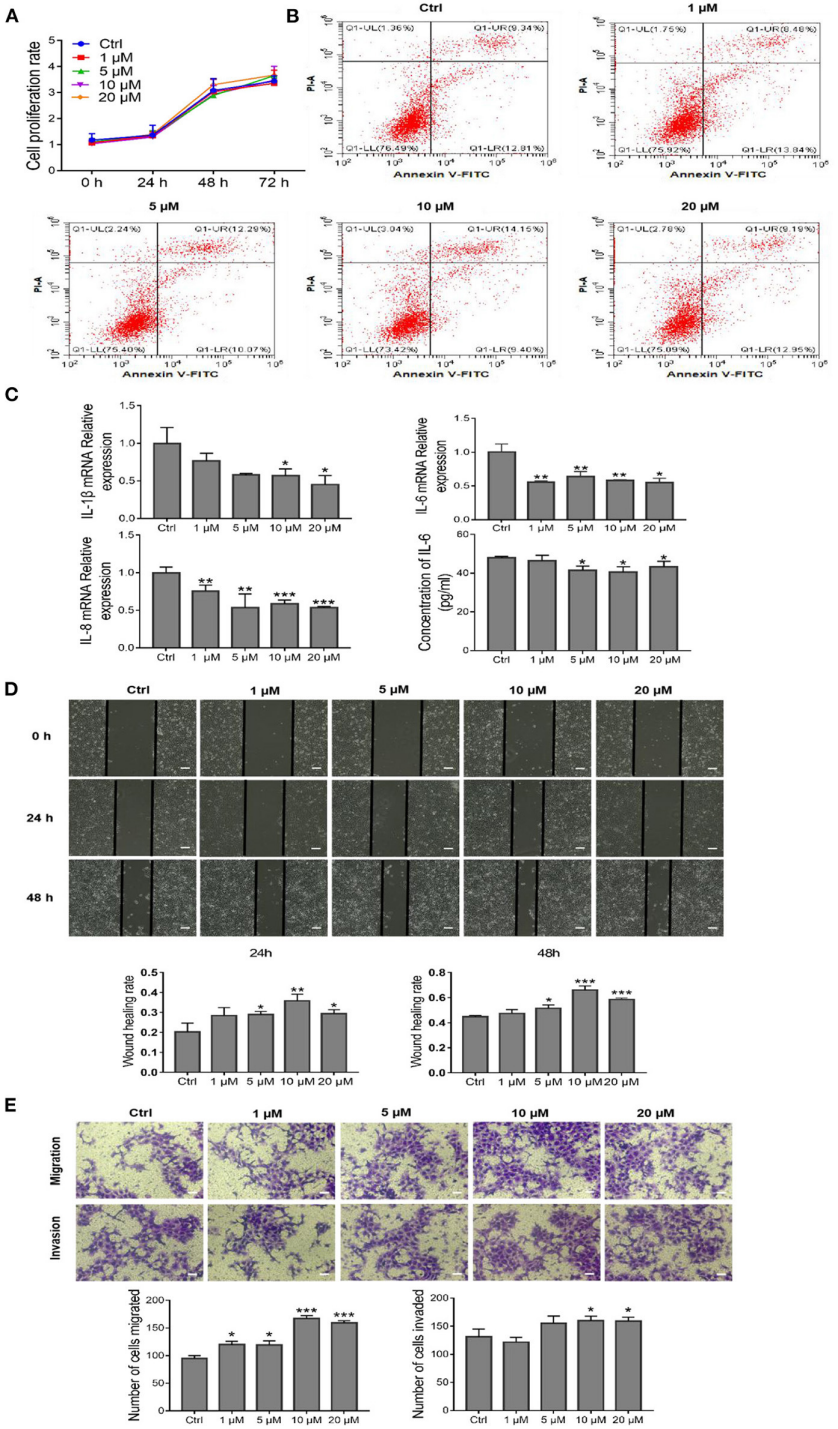
Effects of different concentrations of AEDPPE on HTR-8/SVneo cell function. **(A)** AEDPPE had no significant effect on the proliferation of HTR-8/SVneo cells. **(B)** Different concentrations of AEDPPE did not affect apoptosis of HTR-8/SVneo cells. **(C)** The effects of various concentrations of AEDPPE on the expression levels of IL-1β, IL-6, and IL-8 mRNA and the protein content of IL-6 in the cellular supernatant. **(D)** Wound healing was significantly enhanced by AEDPPE treatment of HTR-8/SVneo cells (scale bar = 400 μm). **(E)** AEDPPE promoted the migration and invasion of HTR-8/SVneo cells (scale bar = 200 μm). *p < 0.05, **p < 0.01, ***p < 0.001 vs. the control group.

To determine the *in vitro* effects of AEDPPE, we treated HTR-8/SVneo cells with different concentrations of AEDPPE (0, 1, 5, 10, and 20 μM). The CCK-8 assay showed that AEDPPE did not exert an effect on the proliferation of HTR-8/SVneo cells (Figure 2A), and flow cytometric results showed that AEDPPE did not affect the apoptotic rate of the various treatment groups (Figure 2B). There was also no significant cytotoxicity of AEDPPE on HTR-8/SVneo cells, which is requisite for its future use as a therapeutic agent. Moreover, AEDPPE significantly downregulated mRNA expression of IL-1β, IL-6, and IL-8 relative to the control. In addition, ELISA of cell supernatant for IL-6 confirmed the superior secretory ability of anti-inflammatory factors due to AEDPPE (Figure 2C).

Therefore, wound-healing and Transwell assays were used to investigate the effects of AEDPPE on HTR-8/SVneo cell migration and invasion. Compared with the control group, the wound-healing rate of HTR-8/SVneo cells was significantly increased after AEDPPE treatment (especially at 10 μM) at 24 and 48 h (Figure 2D). In addition, our results from the Transwell assay were generally consistent with those of the wound-healing assay, as different concentrations of AEDPPE significantly promoted the migration and invasion of HTR-8/SVneo cells (Figure 2E). Collectively, AEDPPE occupies a significant role in suppressing inflammatory factor expression and promoting the migration and invasion of HTR-8/SVneo cells.

### AEDPPE Reverses TNF-α-Induced Injury in HTR-8/SVneo Cells

**Figure 3:**
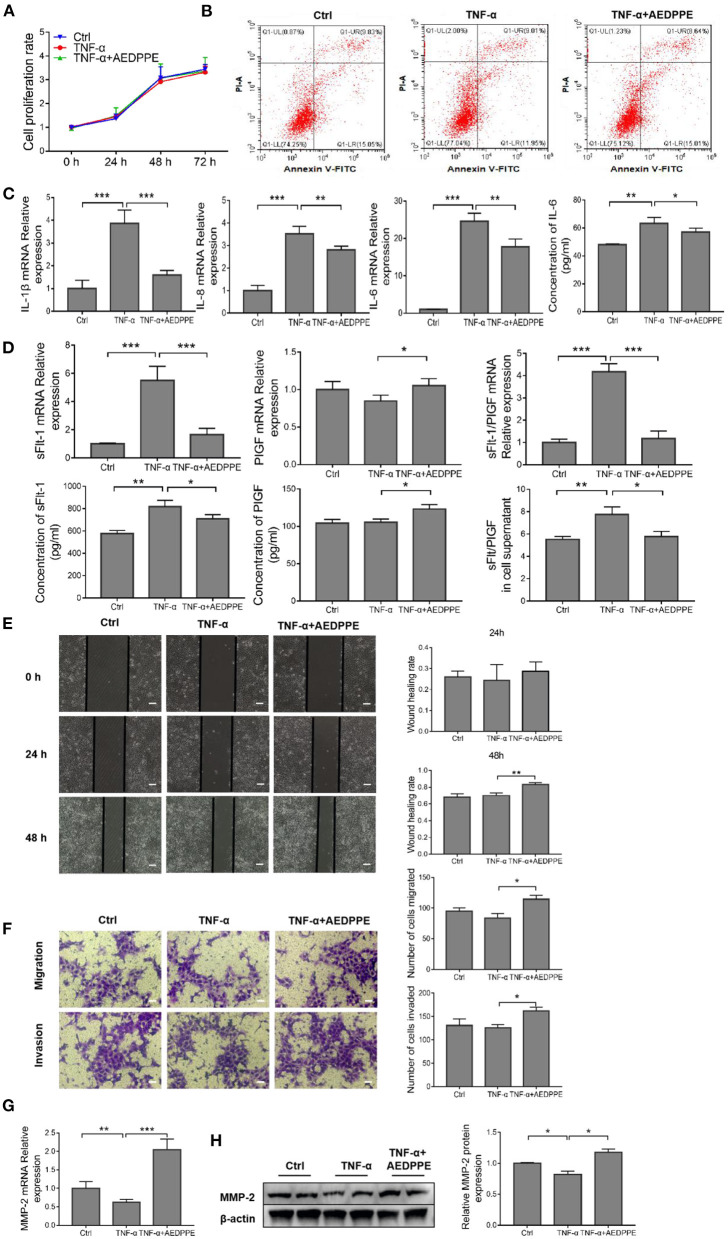
AEDPPE ameliorates TNF-α-induced dysfunction in HTR-8/SVneo cells. **(A)** Treatment of HTR-8/SVneo cells with TNF-α or AEDPPE+TNF-α did not significantly affect cellular proliferation. **(B)** Stimulation with TNF-α and AEDPPE did not affect HTR-8/SVneo cell apoptosis. **(C)** AEDPPE significantly reduced TNF-α-induced mRNA expression of IL-1β, IL-6, IL-8, and IL-6 in cellular supernatants. **(D)** Effects of TNF-α and TNF-α+AEDPPE on mRNA expression of sFlt-1 and PlGF in HTR-8/SVneo cells, and concentrations of sFlt-1 and PlGF protein in cellular supernatant. **(E)** AEDPPE significantly improved wound healing in HTR-8/SVneo cells after TNF-α injury (scale bar = 400 μm). **(F)** AEDPPE promoted migration and invasion of HTR-8/SVneo cells even after TNF-α injury (scale bar = 200 μm). **(G,H)** Effects of TNF-α and TNF-α+AEDPPE on mRNA and protein expression of MMP-2. *p < 0.05, **p < 0.01, ***p < 0.001. IL, interleukin; PlGF, placental growth factor; sFlt-1, soluble FMS-like tyrosine kinase 1; TNF-α, tumor-necrosis factor alpha.

The dysfunction associated with PE *in vivo* was mimicked by the exploitation of an *in vitro* TNF-α-injured HTR-8/SVneo cell model (27–30), and the effects of AEDPPE on cellular proliferation and apoptosis were explored using CCK-8 assays and flow cytometry. We observed no effect on the proliferation or apoptosis of HTR-8/SVneo cells after TNF-α treatment with or without AEDPPE compared to the control group (Figures 3A,B). Although induction of HTR-8/SVneo cell injury with 20 ng/ml of TNF-α did not increase apoptosis, it resulted in significant upregulation of IL-1β, IL-6, and IL-8 mRNA expression. Compared with the TNF-α group, the mRNA expression levels for IL-1β, IL-6, and IL-8 were significantly downregulated in the TNF-α+AEDPPE group. Changes in IL-6 protein content in cellular supernatants between groups were also consistent with changes in mRNA (Figure 3C). Therefore, AEDPPE significantly reduced the expression and secretion of inflammatory factors induced by TNF-α in HTR-8/SVneo cells.

In addition, the mRNA expression levels of sFlt-1 (a predictor of PE) were significantly altered in response to TNF-α, and that the sFlt-1/PlGF ratio was significantly elevated; however, AEDPPE reversed these changes (Figure 3D). We also determined sFlt-1 and PlGF protein alterations in the cellular supernatant by ELISA, which was consistent with the mRNA expression (Figure 3D).

The TNF-α stimulation of HTR-8/SVneo cells for 24 h resulted in a significant decline in the wound-healing area, whereas AEDPPE and TNF-α co-stimulation increased the area after 24 and 48 h (Figure 3E). Similarly, Transwell assays showed that stimulation with AEDPPE improved the migration and invasion of TNF-α-injured HTR-8/SVneo cells (Figure 3F). Using qRT-PCR and Western blotting analyses, we demonstrated that TNF-α significantly downregulated MMP-2 mRNA and protein expression, whereas AEDPPE upregulated its expression; this may be how AEDPPE promoted the migration and invasiveness of HTR-8/SVneo cells (Figures 3G,H). MMP-2, a protein conjectured to influence trophoblast migration and invasion (35), may thus be involved in the biology of such AEDPPE-induced cellular effects on HTR-8/SVneo cells. AEDPPE also showed a singular ability to reverse the damage caused by TNF-α. This then led us to explore the mechanism by which AEDPPE exerted its actions and whether it functioned similarly *in vivo*.

### Improvement of HTR-8/SVneo Cells Function by AEDPPE may Involve the NF-κB Signaling Pathway by Binding to HSP 90β

**Figure 4:**
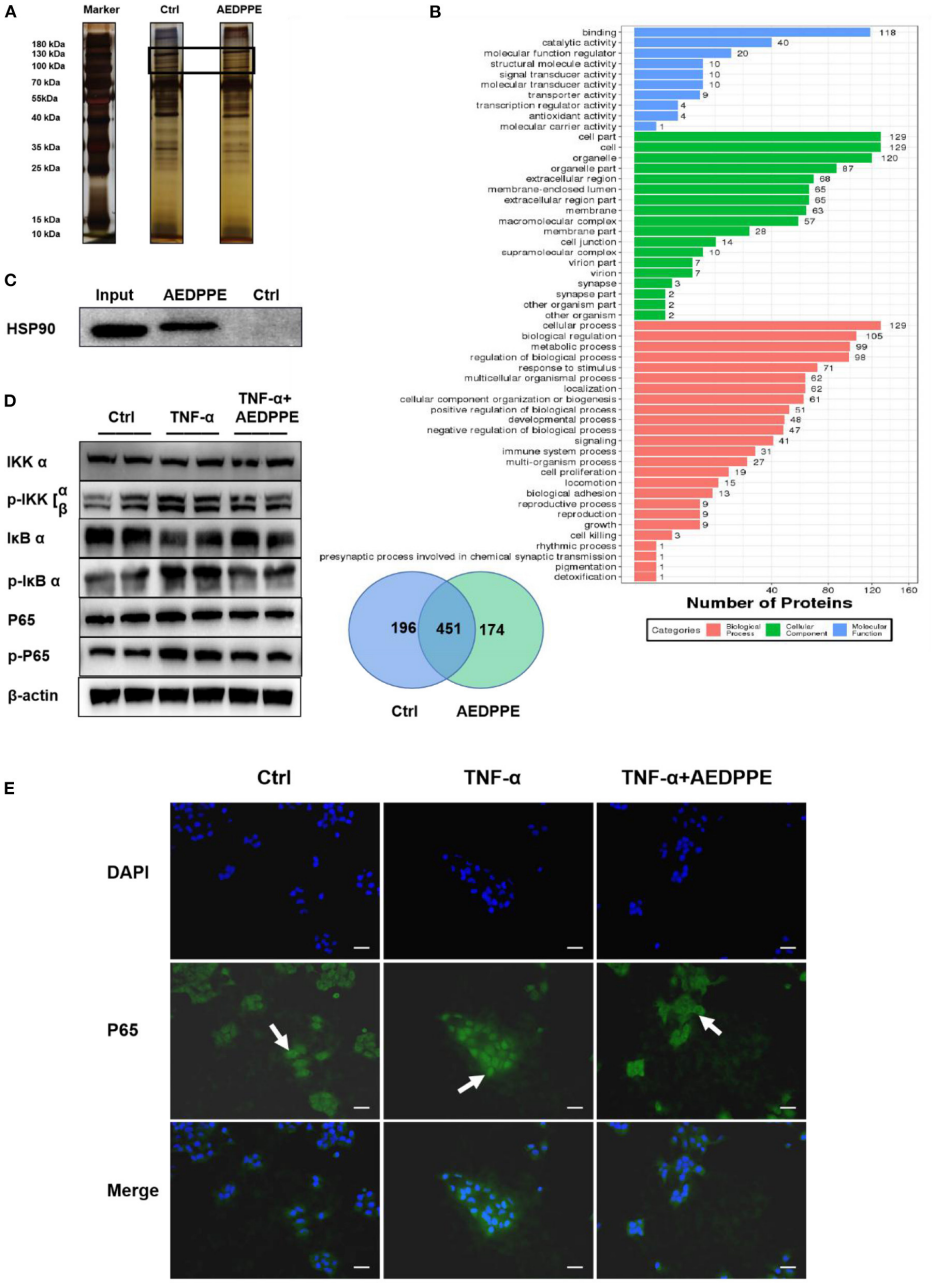
Improvement in HTR-8/SVneo cell function by AEDPPE may involve the NF-κB signaling pathway by binding to HSP 90β. **(A)** Pull-down assay showed that more proteins specifically bound to biotin-AEDPPE at the markers shown compared to those in the control group. **(B)** Gels with differences between the AEDPPE and control groups were analyzed using mass spectrometry and subjected to GO analysis, and we noted protein differences between the two groups. **(C)** AEDPPE bound specifically to HSP 90β *in vitro*. **(D)** TNF-α strongly activated the NF-κB pathway, resulting in increased p-IKK expression, catabolic depletion of IκB α, and increased p-IκB α–ultimately leading to increased expression levels of p-P65. Activation of the NF-κB signaling pathway was inhibited when AEDPPE was co-stimulated with TNF-α compared to TNF-α alone. **(E)** The distribution of P65 (green) in HTR-8/SVneo cells was localized by immunofluorescence. Only unstimulated phosphorylated P65 translocated into the nucleus (blue), although stimulation by TNF-α caused a large amount of P65 to translocate into the nucleus; and P65 in the nucleus was reduced when co-stimulated with TNF-α and AEDPPE (scale bar = 100 μm). IL, interleukin; PlGF, placental growth factor; sFlt-1, soluble FMS-like tyrosine kinase 1; TNF-α, tumor-necrosis factor alpha.

The pull-down assay revealed that AEDPPE bound a variety of proteins (Figure 4A), and mass spectrometric analysis of different portions of the gel identified several proteins that may interact with AEDPPE. Based on the effect of AEDPPE on HTR-8/SVneo cells, we selected HSP 90β from 174 specifically bound proteins in combination with the results of GO analysis of proteins bound to AEDPPE (Figure 4B) and showed that AEDPPE bound HSP 90β *in vitro* (Figure 4C). HSP 90β binds to the HSP 90 co-chaperone Cdc37 (CDC37), which in turn forms a complex with IKK to regulate the IKK/IκB/NF-κB pathway—a process necessary for TNF-α-induced NF-κB activation (36–39). Western blotting then showed that AEDPPE inhibited TNF-α-induced upregulation of p-IKK α/β, p-IκB α, and p-P65 expression (Figure 4D). We further examined the distribution of P65 in the cells by immunofluorescence assays and demonstrated that TNF-α promoted significant aggregation of p-P65 in the nucleus, while AEDPPE inhibited this effect (Figure 4E). The decrease in P65 in the nucleus, therefore, indicated that NF-κB activation was inhibited (40). Binding to HSP 90β and perturbation of the NF-κB pathway may be one of the mechanisms by which AEDPPE reduces inflammatory-factor production and promotes HTR-8/SVneo cell migration and invasion.

### AEDPPE Improves Symptoms in the LPS-Induced PE-Like rat Model

**Figure 5:**
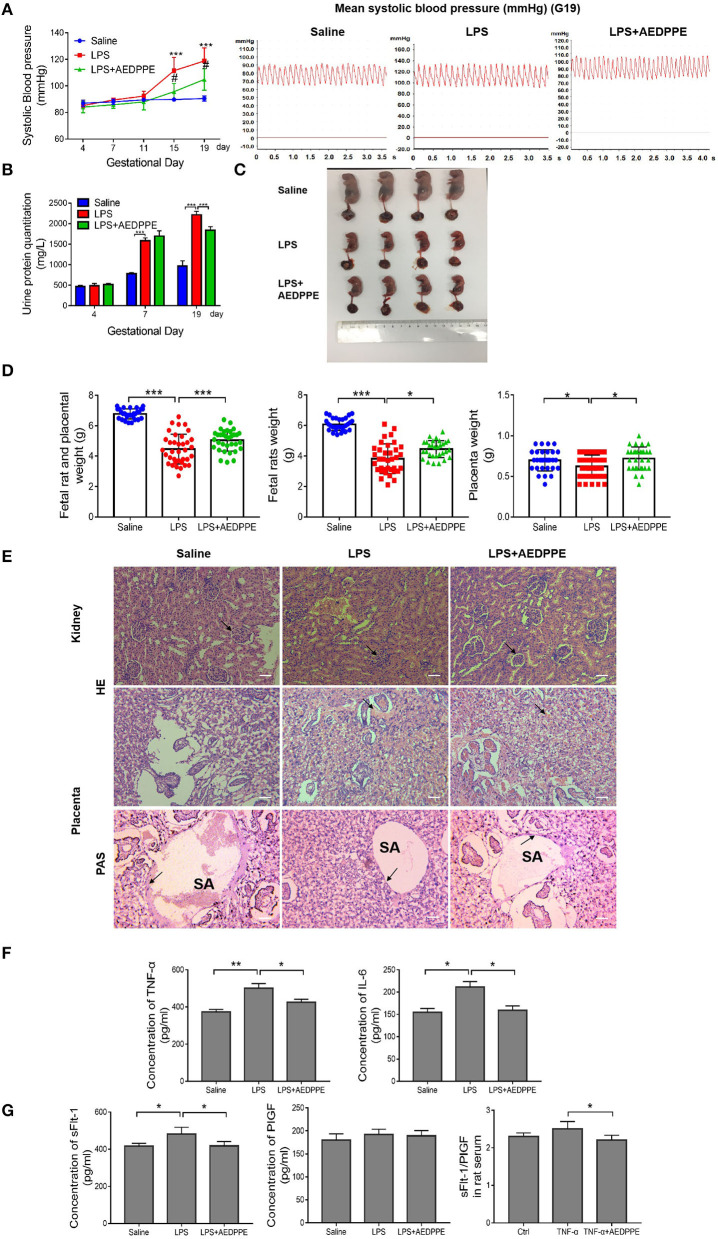
Lipopolysaccharide (LPS) injection causes a variety of symptoms similar to PE, including increased urinary protein, elevated blood pressure, kidney, and placental damage, and very pronounced fetal growth restriction in fetal rats. These symptoms were significantly improved in the LPS+AEDPPE group. **(A)** Blood pressure in rats of the physiological saline group (*n* = 6), LPS group (*n* = 6), and LPS+AEDPPE group (*n* = 6). **(B)** Urine protein levels on day 4, day 7 and day 19 of pregnancy of each group. **(C)** Fetal rats in the LPS group exhibited significant growth restriction compared to those in the saline group. AEDPPE exerted a protective effect against fetal growth restriction. **(D)** Weight of fetal rats and placenta in each group. **(E)** The glomeruli in the LPS group showed mild chronic hyperplasia and swelling compared to the glomeruli of the saline group. Compared to the saline group, H&E staining showed significant vascular congestion in the placental labyrinth, which was improved in the LPS+AEDPPE group. PAS staining in the saline group showed significant fibrin deposition; in contrast, in the LPS-treated group there was little fibrin deposition; and in the LPS+AEDPPE group, a small amount of fibrin could be observed. SA, spiral artery (bar [H&E] = 200 μm, Bar [PAS] = 400 μm). **(F)** Compared with the control group, the serum concentrations of TNF-α (*p* < 0.01) and IL-6 (*p* < 0.05) were significantly higher in the LPS rats; and the concentrations of TNF-α (*p* < 0.05) and IL-6 (*p* < 0.05) were significantly lower in the LPS+AEDPPE group compared with the LPS group. **(G)** The concentrations of sFlt-1 and PlGF in the serum of each group. *p < 0.05, **p < 0.01, ***p < 0.001. H&E, hematoxylin–eosin; IL, interleukin; LPS, lipopolysaccharide; PAS, periodic acid-Schiff; PlGF, placental growth factor; sFlt-1, soluble FMS-like tyrosine kinase 1; TNF-α, tumor-necrosis factor alpha.

To evaluate the protective effect of AEDPPE *in vivo*, rat systolic pressure, urinary protein, placental, and fetal rat weights, and renal and placental pathology were monitored in an LPS-induced PE-like rat model. On days 15 and 19 of gestation, systolic blood pressure in the LPS+AEDPPE group was significantly reduced compared with the LPS group. In addition, we plotted blood pressure waveforms from pregnant rats on day 19 of gestation (Figure 5A), and noted that the LPS-induced upregulation of urine protein levels was significantly reversed with AEDPPE treatment (Figure 5B). The elevated blood pressure and increased urinary protein in rats indicated the success of our PE-like rat model, whereas AEDPPE effectively reduced these symptoms that were induced by LPS.

In addition to the clinical signs in the rats, we executed a preliminary analysis of the fetal conditions of rats. The fetal rats in the LPS group were significantly smaller in size compared with the saline group and showed significant signs of growth restriction (Figure 5C), and placental and fetal rat masses and the sum of fetal rat and placental masses were also significantly reduced (Figure 5D). In the LPS+AEDPPE group, the placental and fetal rat weights, and the combined weight of the two were significantly higher relative to the LPS group.

Furthermore, we analyzed the renal and placental lesions to clarify their damage. Glomeruli in the LPS group exhibited mild chronic hyperplasia and swelling compared to the saline group, but these symptoms were significantly improved in the AEDPPE group. H&E staining of the placental labyrinth area showed that LPS resulted in severe vascular congestion, whereas PAS staining showed that LPS resulted in essentially no fibrin deposition, which may be associated with inadequate remodeling of the spiral arteries. In the LPS+AEDPPE group, we observed reduced vascular congestion and a small amount of fibrin deposition compared to the LPS group (Figure 5E). Compared with the saline group, the concentrations of TNF-α, IL-6, and sFlt-1 were significantly augmented in the serum of LPS rats, whereas, in contrast, the LPS+AEDPPE group showed significantly attenuated concentrations of TNF-α, IL-6, sFlt-1, and sFlt-1/PlGF compared with the LPS group (Figures 5F,G). Collectively, our data revealed that AEDPPE not only improved the clinical signs of the dams, but it also effectively improved the prognosis with respect to the fetal rats.

## Discussion

Preeclampsia causes at least 42,000 maternal deaths per year, with low- and middle-income countries facing the highest burden of major complications (16), and dysfunctional delivery of the placenta currently comprises the only effective and essential treatment (41). In previous studies, researchers have focused on exploring the causes of endothelial damage and how to improve and treat such damage (42, 43), whereas only a few investigators have focused on the impairment and improvement in trophoblast function. Chaudhary et al. (44) found that hepatocyte growth factor modulated migration and invasion of HTR-8/SVneo cells by regulating HIF-1α (44); and Baryla et al. established that prostaglandin F2α promoted adhesion, migration, invasion, and proliferation of HTR-8/SVneo cells (31). Yoshida et al. (45) showed that *Lactobacillus crispatus* promoted HTR-8/SVneo cell invasion *via* activation of MMP-2, and our previous work revealed that such peptides play a significant role in improving trophoblast and umbilical vein endothelial cell function (23).

It has been reported that elevated levels of intra-trophoblastic inflammation and decreased invasion are the principal manifestations of trophoblast dysfunction in PE (46). In our study, 10 μM AEDPPE was shown to significantly promote the migration and invasion of HTR-8/SVneo cells. Other reports revealed that the mRNA expression of IL-1β, IL-6, and IL-8 in HTR-8/SVneo cells was significantly downregulated by AEDPPE. TNF-α is elevated in PE (47), and several studies have shown that TNF-α treatment induced HTR-8/SVneo cell dysfunction—including a diminution in invasive capacity and elevated inflammatory-factor expression—which emulates the dysfunction of PE *in vivo* (48–50). In our study, TNF-α was found to cause significant upregulation of inflammatory-factor expression and impairment of migration and invasion in HTR-8/SVneo cells. Co-stimulation of HTR-8/SVneo cells by TNF-α and AEDPPE resulted in significantly higher migratory and invasive capabilities and lowered expression of inflammatory factors compared to TNF-α treatment alone. In addition, the TNF-α-induced increase in the sFlt-1/PlGF ratio improved after co-stimulation with AEDPPE. In an animal experiment, serum sFlt-1 of the LPS groups was increased, but the change in the sFlt-1/PlGF ratio was not obvious; however, the ratio was significantly reduced in the LPS+AEDPPE group compared with the LPS group. sFlt-1 is an antagonist of vascular endothelial growth factor (VEGF) and PlGF, and its induction of vasoconstriction and endothelial injury may lead to PE (11). The sFlt-1/PlGF ratio also exhibits high predictive significance for PE (51, 52). In Yoshida's study, *Lactobacillus crispatus* precisely promoted the expression and activity of MMP-2 to increase the invasive capacity of HTR-8/SVneo cells, whereas no significant changes were observed in MMP-9 (45). In addition, when the mRNA and protein expression levels of MMP-2 were evaluated, AEDPPE reversed the TNF-α-induced downregulation of MMP-2.

Through pull-down assays combined with mass spectrometry and *in vitro* binding experiments, we uncovered significant coupling of AEDPPE to HSP 90β. HSP 90β regulates the NF-κB pathway by binding to its chaperone protein CDC 37 and then forms a complex with IKK and other molecules (36–39). Our results indicated that AEDPPE inhibited TNF-α-induced NF-κB activation, and immunofluorescence demonstrated that AEDPPE reduced the translocation of P65 into the nucleus. Thus, we speculated that the improvement in HTR-8/SVneo cell function by AEDPPE may involve the NF-κB signaling pathway.

It has been found in numerous studies that LPS leads to the destruction of trophoblast function and activation of the NF-κB pathway in rats, thereby establishing a PE-like rat model (22, 32, 53), and we similarly generated a PE-like rat model by administering LPS to pregnant rats. Twenty-four-hour urinary protein of LPS-stimulated rats was significantly increased on the 7th day of pregnancy, as was systolic blood pressure after the 11th day. By tail-vein injection of AEDPPE, both blood pressure and elevated urinary protein levels were then improved in rats treated with LPS, indicating that the PE status of pregnant rats was improved. Furthermore, in addition to the amelioration of rat symptoms, fetal rat and placental weights and kidney and placental damage were improved. In particular, LPS caused congestion in the labyrinthine area of the placenta and reduced fibrin deposition, whereas AEDPPE reversed these injuries, which may reflect the underlying impetus for the improvement observed in rat symptoms and fetal rat outcome with AEDPPE. Rats in the present study also manifested a significantly belated increase in blood pressure compared with other studies (54), which may relate to the route of administration, the environment in which the rats were housed, and/or the source of the animals. However, increases in blood pressure and urinary protein were sufficient to demonstrate that our PE-like rat model was successfully created and implemented. As fetal growth restriction was evident in our LPS-induced PE model, it is likely that other animal models of PE will be used in future studies to validate the role of AEDPPE.

Trophoblast dysfunction is central to the pathogenesis of PE through the action of TNF-α on trophoblast cells, and LPS-injected rats sustain injuries to trophoblast cells; the activation of NF-κB is then the inevitable result of the action of TNF-α and LPS (27–30, 32). It has been demonstrated in several studies that the NF-κB pathway was robustly activated in patients with PE (12, 13), and the NF-κB pathway has thus been identified as a potential signaling pathway in the treatment of PE by improving trophoblast function or symptoms in animal models (55). Activation of the NF-κB pathway not only promotes the expression of inflammatory factors but also mitigates invasion by trophoblast cells (56, 57). Additionally, we generated predictors of PE by assessing sFlt-1, PlGF, and their ratios. In other animal models of PE, a reduction in circulating sFlt-1 or the administration of exogenous PlGF significantly improved clinical signs (58, 59). We also found that LPS produced an elevation in sFlt-1 concentrations and an increase in the sFlt-1/PlGF ratio in rat serum, whereas AEDPPE reversed both indices. These results indicated the excellent potential of employing AEDPPE in the treatment of PE.

Numerous studies of PE have been conducted using the HTR-8/SVneo cell line (31, 44, 45). However, since a recent study showed that heterogeneous cells exist in the HTR-8/SVneo cell line (60) (which we did not consider in our study), we expect to determine the effects of AEDPPE in the future using different trophoblast cell lines.

Whether the binding of AEDPPE to HSP 90β directly affected the binding of HSP 90β to CDC 37 or the formation of complexes with IKK was not determined in the present study. Although we found that the downstream targets of NF-κB signaling (such as IL-6) were altered upon AEDPPE treatment, the *in vivo* effect of AEDPPE on the NF-κB signaling pathway remains arcane. We will, therefore, address these investigational shortcomings in future studies. In summary, our preliminary exploration revealed that AEDPPE constitutes a promising therapeutic agent in the treatment of PE.

## Data Availability Statement

The original contributions presented in the study are included in the article/supplementary materials, further inquiries can be directed to the corresponding author/s.

## Ethics Statement

The animal study was reviewed and approved by Ethics Committee of Nanjing Medical University (Approval No: 2005040).

## Author Contributions

YW and YC: design of experiments, conduct of experiments, data analysis, and writing of the manuscript. XJ and CL: design and conduct of experiments, data analysis, and guidance on manuscript revision. LX and TL: design and guidance of experiments and data analysis. HD and RJ: design and guidance of experiments, editorial review of the manuscript, and financial support. All authors contributed to the article and approved the submitted version.

## Funding

This research was financially supported by the National Natural Science Foundation of China (81771604, 81801470) and Jiangsu Provincial Key Research and Development Program (Grant No. BE2021614).

## Conflict of Interest

The authors declare that the research was conducted in the absence of any commercial or financial relationships that could be construed as a potential conflict of interest.

## Publisher's Note

All claims expressed in this article are solely those of the authors and do not necessarily represent those of their affiliated organizations, or those of the publisher, the editors and the reviewers. Any product that may be evaluated in this article, or claim that may be made by its manufacturer, is not guaranteed or endorsed by the publisher.
